# Effects of methyl salicylate pre-treatment on the volatile profiles and key gene expressions in tomatoes stored at low temperature

**DOI:** 10.3389/fnut.2022.1018534

**Published:** 2022-10-05

**Authors:** Xiangquan Zeng, Libin Wang, Yingli Fu, Jinhua Zuo, Yan Li, Jingling Zhao, Rui Cao, Jian Li

**Affiliations:** ^1^Department of Food Quality and Safety, School of Food and Health, Beijing Engineering and Technology Research Center of Food Additives, Beijing Technology and Business University, Beijing, China; ^2^School of Light Industry and Food Science, Nanjing Forestry University, Nanjing, Jiangsu, China; ^3^Beijing Vegetable Research Center, Beijing Academy of Agriculture and Forestry Science, Beijing, China

**Keywords:** methyl salicylate, low temperature, tomato, volatile biosynthetic pathways, flavor compounds

## Abstract

Tomato is one of the most widely cultivated horticultural plants in the world, while the key volatile compounds of tomato fruits generally derive from fatty acid, carotenoid, phenylalanine, and branched-chain amino acid pathways. As an important endogenous signal molecule, methyl salicylate (MeSA) plays a crucial role in the fruit ripening process of plant. Recently, it has been demonstrated that MeSA can maintain the flavor quality of full ripe tomatoes after cold-storage preservation. However, few research teams attempted to investigate the effects of MeSA plus low temperature treatment on the different volatile biosynthetic pathways of tomatoes previously. Therefore, in this study, the effects of methyl salicylate pre-treatment (0.05 mM MeSA, 24 h) on the volatile profile and flavor-related key gene expressions of tomato fruits stored at 10°C were evaluated for the first time. Our results showed that the loss of volatile compounds in low temperature-treated tomato fruits could be effectively alleviated by MeSA pre-treatment. Although MeSA had no remarkable effect on the formation of carotenoid pathway- and branched-chain amino acid pathway-related volatiles in tomatoes subjected to low temperature, the content of fatty acid pathway-related volatiles (including *cis*-3-hexenal, hexanal, and *trans*-2-hexenal) in full red fruits of 10°C MeSA group was remarkably higher than that of 10°C control group. Furthermore, MeSA pre-treatment significantly up-regulated the expression of *LOXC* or *LOXD* gene in low temperature-treated fruits at breaker or full red stage, respectively. In conclusion, pre-treatment with MeSA might avoid the loss of aromatic compounds in tomato fruits stored at low temperature by activating the fatty acid pathway.

## Introduction

Tomato is one of the most widely cultivated horticultural plants in the world, which accounts for 23% of total output in the whole fruit and vegetable market (1, 2). It has been demonstrated that tomato fruits are rich in vitamins, flavonoids, carotenoids, and other bioactive compounds (3). As an important sensory quality parameter for tomato fruit, the aroma is closely associated with the consumers’ acceptance of tomatoes. Nowadays, over 400 volatile compounds have been identified in tomatoes, including primary aromatic compounds and a series of secondary aromatic compounds (4). Among them, aldehydes, alcohols, ketones, esters, phenols, and sulfurs play important roles in the flavor of tomatoes.

The key volatile compounds of tomato fruits usually derive from fatty acid, carotenoid, phenylalanine, and branched-chain amino acid pathways (5). It is reported that C6-aroma compounds can be synthesized from fatty acid pathway in tomato fruits, which are a large group of flavor substances with grassy and green odors, including hexanal, *cis*-3-hexenal, and *trans*-2-hexenal (6–8). Specifically, linolenic acid or linoleic acid is catalyzed by lipoxygenases (*LOX*s) and hydroperoxide lyase (*HPL*) to form hexanal or *cis*-3-hexenal, respectively. Under the action of aldehyde dehydrogenase 2 (*ADH2*), these compounds can be reduced to the corresponding alcohols (4). Furthermore, the aromatic compounds with fruity flavors such as neral, geranial, geranylacetone, 6-methyl-5-hepten-2-one, β-ionone, are derived from β-carotene precursors via carotenoid pathway in tomatoes. Carotenoids are able to be degraded by the carotenoid cleavage dioxygenase (*LeCCD*s) to form primary oxidation products, which further transform into volatile compounds under the actions of enzyme catalysis and acid hydrolysis (9).

Also, the volatiles synthesized from phenylalanine pathway have significant effects on the overall flavor of tomato fruits, containing 2-phenylacetaldehyde, 2-phenylethanol, methyl salicylate, guaiacol, eugenol, catechol, 1-nitro-2-ethylbenzene, and 2-phenylacetonitrile (10). On the one hand, phenylalanine can be catalyzed by phenylalanine ammonia lyase (*PAL*) to form *trans*-cinnamic acid, which further passes through different metabolic branches to form salicylic acid. One the other hand, amino acid decarboxylase (*LeAADC*) is able to promote the transformation of phenylalanine into phenylethylamine (11). Alternatively, the formation of aromatic compounds with floral smell (e.g., 2-phenylacetaldehyde and 2-phenylethanol) are related with the effects of *LeAADC* and phenethylamine reductase (*PAR*) (12). Regarding the branched-chain amino acid pathway, isoleucine and leucine are the representative precursors of aromatic components with caramel flavor (e.g., 2-methylbutyraldehyde, 3-methylbutyraldehyde, 2-methylbutanol, and 3-methylbutanol), fruity and spicy flavor (e.g., isovaleronitrile and isobutyl acetate) as well as green and fruity flavor (e.g., 2-isobutylthiazole). Notably, branched-chain amino acid aminotransferases (*SlBCAT*s) are essential for the synthesis of these components (13).

Based on previous studies, the flavor of tomato fruits can be significantly influenced by their varieties and maturity stages as well as different postharvest treatments (including ethylene, low temperature, and hormone treatments) (14). Cold storage is shown to be one of the most effective postharvest treatment methods to prolong the shelf life of horticultural products (15). However, it may inhibit the flavor formation of tomato fruits. For instance, Wang et al. (6) investigated the changes of volatile compounds in tomatoes at the green ripening stage under low temperature (5°C) (6). The results showed that the level of twelve important compounds (including aldehydes and alcohols) in tomato fruits significantly decreased after treating under low temperature. In addition, the ripening process, ethylene release, and the respiratory rate of tomatoes were also inhibited by low temperature treatment although no obvious mechanical injury was observed. It was worth mentioning that the volatile content of fruits stored at 5°C was at a very low level before the color-breaking stage. In the research of Ponce-Valadez et al. (16), they observed that storing at 12.5°C over 9 days could lead to a decrease of total aroma volatiles in tomatoes, especially for hexanal, hexanol and *cis*-3-hexenol (16). Nevertheless, there was no significant difference in consumer’s flavor perception between the fruits treated at 12.5 and 20°C.

Methyl salicylate (MeSA), a plant volatile organic compound, is synthesized from salicylate acid (SA) and essential for the growth, development as well as functions of plants (17). As an important endogenous signal molecule, MeSA plays a crucial role in defense mechanism activation, responses against several abiotic and biotic stresses as well as the fruit ripening process (18). In recent years, postharvest treatment with MeSA has drawn increasing interest due to its effects on extending the shelf-life of fresh agricultural products and reducing their low-temperature susceptibility (19). For instance, the chilling injury symptoms of tomatoes exposed to 0.01 mM of MeSA for hours at room temperature were significantly less severe than those of control (20). Interestingly, in a previous investigation, it was found that MeSA pre-treatment could effectively improve the flavor quality of full ripe “FL 47” tomatoes after cold storage as well (6). To be specific, MeSA remarkably suppressed the loss of some key aroma volatiles (including geranylacetone, geranial, and MeSA) in tomato fruits subjected to chilling temperature. However, little information about the effects of MeSA plus low temperature treatment on the volatile biosynthetic pathways (fatty acid, carotenoid, phenylalanine, and branched-chain amino acid pathways) of tomato fruits was provided. Consequently, we aimed to explore the roles of MeSA pre-treatment in the flavor components and flavor-related key gene expressions of tomatoes stored at low temperature in the present study.

## Materials and methods

### Plant materials

Mature green (G) “FL 47” tomatoes were harvested from a commercial field in Fort Pierce, FL. In the current research, fruits with moderate size and no obvious mechanical damage were selected and used in the following experiment.

### Methyl salicylate plus low temperature treatment

One hundred and forty-four uniform and defect-free fruits with an average weight of 270 g were divided into two groups. Half of them were treated with MeSA at 20°C for 24 h, while the left fruits were stored in air. In terms of the MeSA treated group, the fruits were placed in a 45 L airtight glass container, from the top of which a 7 cm diameter filter paper disc soaked in 222.9 μL of MeSA was suspended. The final chemical vapor concentration in the container was 0.05 mM. After fumigating for 24 h at 20°C, the container was opened, and ventilated for 12 h. Subsequently, all fruits were further classified into four groups (36 fruits each group), respectively. To be specific, 72 fruits with and without MeSA pre-treatment were stored at 20°C for ripening, while another half were transferred to 10°C for 10 days before ripening at 20°C. The samples at G stage (0 day) and other three maturity stages [full red (R), pink (P), and breaker (BR) stages] were collected in this study. For 20°C control group, tomato fruits reached to BR, P or R stage on the 4th, 10th, or 14th day after MeSA pre-treatment, respectively. By contrast, it took 5, 11, or 14 days for fruits in the 20°C MeSA group to reach to BR, P or R stage after MeSA pre-treatment, respectively. The ripening of fruits in 10°C control and 10°C MeSA groups was significantly delayed by low temperature treatment. Particularly, tomatoes in 10°C control group reached to BR, P, or R stage on the 13th, 22nd, or 28th day after MeSA pre-treatment, respectively, whilst it took 13, 21, or 27 days for the fruits of 10°C MeSA group to reach the corresponding stage after treating with MeSA.

### Volatile compound analysis

Volatile compound analysis was performed using gas chromatography-olfactometry-mass spectrometry (GC-O-MS) according to the method of Li et al. (21), with some modifications (21). Briefly, a sharp stainless steel knife was used to remove the peels from three tomato fruits at the same maturation stage per replicate, then the pulp tissues was squeezed into juice (Baijie, S-308, China). Subsequently, 10.0 g of juice was mixed with 1.0 g of NaCl solution and immediately transferred to a 40 mL headspace bottle, which was incubated in a 60°C thermostatic water bath for 10 min to activate volatile compounds. Volatiles compounds were separated by a DB-WAX capillary column (30 m × 0.25 mm × 0.25 μm) and further identified by 7890B Agilent GC coupled to 5977A mass spectrometer detector (Agilent Technologies, USA). The temperature programming was as follows: The initial temperature (35°C) was held for 0 min before increasing to 180°C at a rate of 3°C/min, then it ramped to 230°C at a rate of 15°C/min and maintained for 2 min. High purity helium was utilized as the carrier gas at a flow rate of 1.3 mL/min, while electron impact ionization at electron energy of 70 eV and a mass range of m/z 50–500 amu were used for MS.

Three assessors trained over 2 weeks were employed to conduct GC-O-MS analysis. The panel members determined aroma intensities using a ten-point intensity scale, wherein 1, 5, and 10 corresponded to weak intensity, moderate intensity, and extreme intensity, respectively. Each sample was sniffed by assessor panelists twice, and the parameters such as aroma descriptions, retention time and intensity value were recorded. The peak was considered the active aroma only if at least two experimenters found similar odor at the same retention time.

### RNA isolation and cDNA preparation for expression analysis

The total RNA was isolated from the pulp of tomatoes based on the method described by Singh et al. (22). Subsequently, the reversed transcription of RNA to cDNA was performed using the Revert Aid First-Strand cDNA Synthesis Kit (Fermentas, Madison, WI, USA) in accordance with the manufacturer’s instructions. To verify the expression patterns revealed by the RNA-seq technology, quantitative real-time PCR (qRT-PCR) analysis was conducted in our research. Genes tested included *LOX*s, *ADH2*, *HPL*, *LeCCD*s, and *SlBCATs*. Gene specific primers for selected genes were designed by online software,^1^ while the melt curve was used to analyze the amplification curve specificity. Besides, qRT-PCR analysis was performed using Fast SYBR Mixture on a Bio-Rad CFX connected with real-time PCR detection system, and the incubation conditions of test was based on a two-step method: 95°C for first 10 min, followed by 40 cycles of 95°C for 15 s and 60°C for 60 s. Relative expression level was calculated based on the 2^–Δ^ ^Δ^ *^Ct^* method using actin as an internal reference gene. Three biological replicates were used for all qRT-PCR experiments and average data from three was plotted.

### Statistical analysis

The volatile compounds were identified by comparing their retention indexes (RI) and mass−fragmented patterns with standards, mass spectra in the NIST Database, and/or previously published studies (23). All the experiments were performed by triplicate assays, and the results were expressed as the means ± standard deviation (SD) using SPSS 19.0 (USA) for windows. The statistical significances of data were determined using one-way analysis of variance (ANOVA) followed by the comparison of Duncan’s Multiple Range Test (DMRT), *p*-values < 0.05 were regarded as significant.

## Results and discussion

### The volatile profiles and key gene expressions of tomato fruits stored at 20°C

**FIGURE 1:**
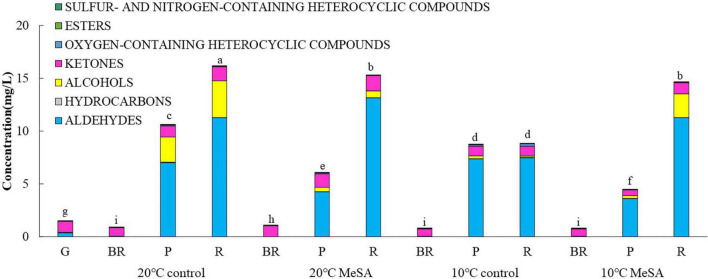
Effects of methyl salicylate (MeSA) on the contents of different types of volatile compounds in low temperature-treated tomato fruits. Data are expressed as mean ± SD (*n* = 3), repeated measures one-way ANOVA followed by Duncan’s multiple range test (DMRT). Data marked with the same letter were no significant difference at *p* < 0.05.

As shown in Table 1, a total of 37 volatile compounds in “FL 47” tomato fruits were identified by GC-O-MS analysis, containing 16 aldehydes, five alcohols, five ketones, two esters, four hydrocarbons, three oxygen-containing heterocyclic compounds, as well as two sulfur and nitrogen-containing heterocyclic compounds. The findings were similar to those of Wang et al. (6), they identified 42 volatile compounds in full ripe “FL 47” tomatoes by headspace-solid-phase micro-extraction-GC-MS (HS-SPME-GC-MS) analysis (6). It was shown in Figure 1 that the content of aldehydes was the highest in volatile compounds of most groups and their flavors were described as green. As the tomato fruits ripened, the aldehyde content significantly increased. In line with Xi et al. (24), *cis*-3-hexenal was the most predominant compound among 16 aldehydes (24), while its percentage in total volatiles of tomatoes at P or R stage without any treatment was 38.48 or 69.89%, respectively (Table 2).

**TABLE 1:** The retention index and odor descriptions of the volatile compounds identified by the GC-MS analysis.

Volatile compounds	Retention index	Odor description
**Aldehydes**		
Butanal	590	Pungent, green
Isovaleraldehyde	890	Malt
2-Methylbutanal	647	Malt
Tiglic aldehyde	1,101	Green, fruit
*trans*-2-Pentenal	828	Strawberry, fruit, tomato
*cis*-3-Hexenal	771	Leafy, green
Hexanal	773	Grass, tallow, fat
*trans*-2-Hexenal	828	Green, leafy
Heptanal	875	Fat, citrus
*trans, trans*-2, 4-Hexadienal	886	Green
Benzaldehyde	948	Almond, burnt sugar
Octanal	970	Fat, soap, green
Benzeneacetaldehyde	1,062	Honey, sweet
2-Octenal	987	Green leafy, walnut
Non-anal	1,059	Fat, citrus, green
**Hydrocarbons**		
α-Pinene	910	Pine, turpentine
*p*-Cymene	994	Solvent, gasoline, citrus
_*D*_-Limonene	998	Lemon, orange
Terpinolene	1,049	Pine, plastic
**Alcohols**		
2-Methylpropanol	614	Wine, solvent, bitter
3-Methylbutanol	707	Whiskey, malt, burnt
2-Methylbutanol	711	Malt, wine, onion
1-Pentanol	1,107	Balsamic
3-Methylpentanol	817	Pungent
**Ketones**		
Acetone	534	Pungent, irritating, floral
2-Butanone	592	Sweet
1-Penten-3-one	661	Fruity, floral, green
6-Methyl-5-hepten-2-one	951	Fruity, floral
Geranyl acetone	1,368	Sweet, floral, estery
**Oxygen-containing heterocyclic compounds**		
2-Methylfuran	595	Chocolate
2-Ethyl furan	675	Rum, coffee and chocolate
2-pentyl-furan	996	Green bean, butter
**Esters**		
Butyl acetate	759	Pear
2-Methylbutyl acetate	866	Fruit
**Sulfur- and nitrogen-containing heterocyclic compounds**		
2-Isobutylthiazole	1,002	Tomato leafy, green
Dimethyl-disulfide	1,071	Onion, cabbage, putrid

**TABLE 2:** Effects of methyl salicylate (MeSA) pre-treament on the volatile profiles of tomato fruits stored at low temperature.

Concentration (mg/L) compounds		20°C control			20°C MeSA			10°C control			10°C MeSA		
	
	G	BR	P	R	BR	P	R	BR	P	R	BR	P	R
**Aldehydes**													
Butanal	-	-	0.00379^bc^	0.01009^a^	-	0.00514^b^	0.00797^a^	-	0.00060^d^	0.00163^cd^	-	-	0.00375^bc^
Isovaleraldehyde	0.001172^e^	-	0.68416^c^	0.85113^ab^	-	0.78050^ac^	0.85590^a^	-	0.22263^d^	0.86813^a^	-	0.06435^e^	0.74258^bc^
2-Methylbutanal	-	-	-	-	-	-	-	-	-	-	-	-	-
Tiglic aldehyde	-	-	0.65628^b^	0.91665^a^	-	0.69762^b^	0.80697^ab^	-	-	0.72970^b^	-	-	0.72407^b^
*trans*-Pentenal	-	-	-	0.00927^b^	-	-	0.02077^a^	0.00318^c^	-	-	-	-	0.01319^b^
*cis*-3-Hexenal	-	-	4.08108^bd^	7.29117^ab^	-	1.93157^de^	8.87602^a^	-	5.69925^ac^	4.45166^bd^	-	2.70142^ce^	7.41624^ab^
Hexanal	0.367712^de^	-	1.25997^ac^	1.55635^ac^	-	0.73528^ce^	1.71419^a^	-	1.17889^ad^	1.12850^ad^	-	0.82166^be^	1.63075^ab^
*trans*-2-Hexenal	-	-	0.37929^cd^	0.63588^ac^	-	0.09721^d^	0.81403^a^	-	0.27169^cd^	0.29695^cd^	-	0.00274^d^	0.72669^ab^
Heptanal	-	-	0.00467^c^	0.01183^a^	-	0.00573^c^	0.01260^a^	-	0.00195^de^	0.00422^cd^	-	-	0.00824^b^
*trans, trans*-2, 4-Hexadienal	- -	-	-	-	-	-	-	0.00624	-	-	-	-	-
Benzaldehyde	-	-	0.00052^c^	0.00106^b^	-	0.00058^c^	0.00117^b^	-	-	0.00031^d^	-	-	0.00139^a^
Octanal	-	0.00060^a^	-	-	-	-	-	0.00054^a^	-	-	-	-	-
Benzeneacetaldehyde	-	-	-	-	-	-	0.05162	-	-	-	-	-	-
2-Octenal	-	-	-	-	-	-	0.00523	-	-	-	-	-	-
Non-anal	0.000119^c^	0.00032^a^	-	0.00011^cd^	0.00018^b^	-	0.00010^d^	-	-	-	-	-	-
**Hydrocarbons**													
α-Pinene	0.000112^de^	0.00024^ae^	0.00016^ce^	0.00011^de^	0.00026^a–d^	0.00010^de^	0.00008^de^	0.00036^ab^	0.00006^e^	0.00042^a^	0.00021^b–e^	0.00012^de^	0.00031^a–c^
*p*-Cymene	-	0.00015^bc^	-	0.00142^a^	0.00021^b^	-	-	0.00002^cd^	-	-	0.00013^b–d^	0.00003^cd^	-
*_*D*_*-Limonene	0.50085^b^	-	0.02252^de^	0.01393^ef^	-	0.01398^ef^	0.01192^f^	0.03462^c^	0.01967^df^	0.06383^a^	0.01259^ef^	0.02425^d^	0.02895^cd^
Terpinolene	-	-	-	0.00062^a^	-	-	0.00002^bc^	-	-	0.00008^b^	-	-	0.00005^bc^
**Alcohols**													
2-Methylpropanol	-	-	0.01730^c^	0.03770^b^	0.00108^g^	0.01204^d^	0.04019^a^	-	0.00348^e^	0.00357^e^	-	-	0.00318^f^
3-Methylbutanol	-	-	1.85744^c^	2.72023^a^	-	1.91057^c^	2.40270^b^	-	0.93333^d^	1.72721^c^	-	0.06917^e^	1.84948^c^
2-Methylbutanol	-	0.01592^g^	0.47700^c^	0.64783^a^	-	0.42209^d^	0.57772^b^	-	0.20312^f^	0.36492^e^	-	0.18759^f^	0.34749^e^
1-Pentanol	-	-	-	-	-	-	-	-	-	-	-	-	-
3-Methylpentanol	-	-	0.03911^b^	0.06448^a^	-	-	0.03954^b^	-	0.03455^b^	0.03783^b^	-	-	0.02846^b^
**Ketones**													
Acetone	1.040855^cd^	0.81874^def^	1.07578^bcd^	1.31415^ab^	1.02152^cd^	1.22290^abc^	1.47779^a^	0.68821^ef^	0.95501^cde^	0.88831^de^	0.72412^ef^	0.56623^f^	1.04710^cd^
2-Butanone	-	-	0.00284^d^	0.00757^a^	-	0.00386^c^	0.00598^b^	-	0.00045^ef^	0.00122^e^	-	-	0.00281^d^
1-Penten-3-one	-	-	-	-	-	-	-	-	-	-	-	-	-
6-Methyl-5-hepten-2-one	-	-	0.01048^b^	0.02199^a^	-	0.01978^a^	-	-	0.00017^b^	0.02246^a^	-	-	0.00890^b^
Geranyl acetone	-	-	-	-	-	-	-	-	-	-	-	-	-
**Oxygen-containing heterocyclic compounds**													
2-Methylfuran	0.000673^c^	0.00026^d^	0.00073^c^	0.00103^b^	0.00068^c^	0.00103^b^	0.00199^a^	-	0.00016^de^	0.00013^de^	-	0.00007^de^	-
2-Ethyl furan	-	0.00749^ab^	0.00771^ab^	0.00974^a^	0.00968^a^	0.00607^ab^	0.00783^ab^	0.01028^a^	0.00866^ab^	0.00528^ab^	0.00819^ab^	0.00793^ab^	0.00820^ab^
2-pentyl-furan	-	0.00163^g^	0.07296^d^	0.01794^f^	-	0.11360^b^	-	0.00245^g^	0.09487^c^	0.21697^a^	0.00205^g^	0.00255^g^	0.03856^e^
**Esters**													
Butyl acetate	-	-	0.00205^c^	0.00259^b^	-	0.00360^a^	0.00262^b^	-	0.00066^d^	0.00257^b^	-	-	0.00072^d^
2-Methylbutyl acetate	-	0.00008^h^	0.00006^i^	-	0.00124^a^	0.00041^f^	0.00061^e^	0.00068^d^	0.00074^c^	0.00097^b^	0.00028^g^	-	-
**Sulfur- and nitrogen-containing heterocyclic compounds**													
2-Isobutylthiazole	-	-	-	-	-	-	-	0.00131^a^	-	-	-	0.00083^b^	-
Dimethyl-disulfide	0.00128^c^	-	-	-	-	-	-	-	0.00661^a^	-	-	0.00203^b^	-
**Total volatiles**	1.462008	0.84543	10.6059	16.14487	1.03845	6.06163	15.29267	0.74789	8.69958	8.813554	0.74757	4.4509	14.63111

Data are expressed as mean ± SD (*n* = 3), repeatedmeasures one-way ANOVA followed by Duncan’s test formultiple comparisons. Datamarked with the same letter were no significant difference at *P* < 0.05.

Furthermore, the alcohols or ketones were the second most abundant volatile compounds, the odor of which was described as malt or flora, respectively. However, the variation trend of alcohol and ketone contents with maturity was different from that of aldehyde content. The alcohol level in flavor components of fruits without any treatment continuously increased prior to the P stage, while decreased at R stage. By contrast, the concentration of ketones at BR, or P stage was relatively lower than those at G and R stage. It might be attributed to the difference in the biosynthetic pathways of these aromatic compounds (22). It was worthwhile to note that 3-methylbutanol or acetone was the major alcohol or ketone compound in volatiles of the control, respectively. When the fruit was at R stage, the level of 3-methylbutanol or acetone could reach to 16.85 or 8.14%. On the basis of the data of volatile composition, green and malt might be the main flavors in the whole odor description of tomato fruits (Table 1).

**FIGURE 2:**
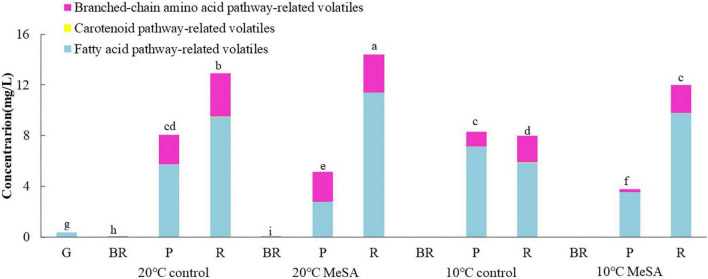
Effects of methyl salicylate (MeSA) pre-treatment on the levels of fatty acid pathway-, carotenoid pathway-, and branched-chain amino acid pathway-related volatiles in tomato fruits stored at low temperature. Data are expressed as mean ± SD (*n* = 3), repeated measures one-way ANOVA followed by Duncan’s multiple range test (DMRT). Data marked with the same letter were no significant difference at *p* < 0.05.

To investigate the effects of postharvest treatments on the volatile biosynthetic pathways of tomatoes, the volatile compounds were divided into four groups in the current research as well, including fatty acid pathway- (*cis*-3-hexenal, hexanal, trans-2-hexenal, 1-pentanol, and 1-penten-3-one), carotenoid pathway- (6-methyl-5-hepten-2-one and geranyl acetone), phenylalanine pathway- (MeSA) and branched-chain amino acid pathway-related volatiles (2-methylbutanal, 3-methylbutanal, 2-methylbutanol, and 2-isobutylthiazole) (25). It could be found in Figure 2 that no MeSA was detected in the volatiles of tomato fruits, possibly due to the difference in the analytic method utilized in the present study. In addition, fatty acid pathway- and branched-chain amino acid pathway-related volatiles played more important roles in the aromatic compounds of tomato fruits in 20°C control group than carotenoid-related volatiles, while their levels gradually increased with the ripening of fruits. The contents of fatty acid pathway-related volatiles in fruits at P and R stages were significantly higher than those of other volatiles, while branched-chain amino acid pathway-related volatiles were the main components of aromatic compounds in tomatoes at BR stage. Additionally, the concentration of fatty acid pathway-related volatiles in fruits of 20°C control group at R stage was approximately 2.78-fold that of branched-chain amino acid pathway-related volatiles. Hence, it seemed that the fatty acid and branched-chain amino acid pathways could be greatly activated to promote the formation of relevant flavor components when the tomatoes ripened.

**FIGURE 3:**
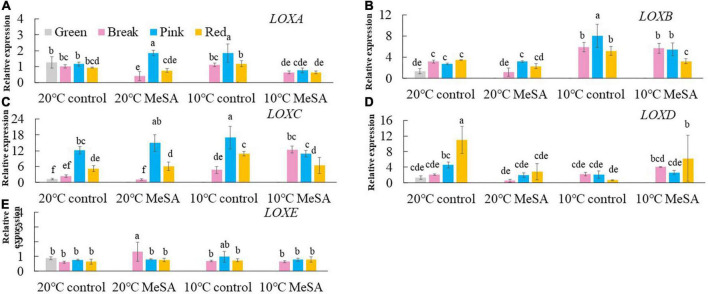
Effects of methyl salicylate (MeSA) pre-treatment on the relative expressions of *LOXA* **(A**), *LOXB* **(B)**, *LOXC* **(C)**, *LOXD* **(D)**, and *LOXE* **(E)** genes in tomato fruits stored at low temperature. Data are expressed as mean ± SD (*n* = 3), repeated measures one-way ANOVA followed by DMRT. Data marked with the same letter were no significant difference at *p* < 0.05.

**FIGURE 4:**
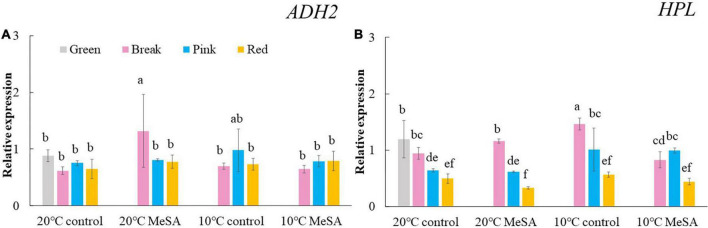
Effects of methyl salicylate (MeSA) pre-treatment on the relative expressions of *ADH2* **(A)** and *HPL* **(B)** genes in tomato fruits stored at low temperature. Data are expressed as mean ± SD (*n* = 3), repeated measures one-way ANOVA followed by DMRT. Data marked with the same letter were no significant difference at *p* < 0.05.

A series of key genes were selected to further verified the data obtained in volatile analysis, such as *LOX*s, *ADH2*, *HPL*, *LeCCD*s, and *SIBCAT*s. *LOX*s (*LOXA*, *LOXB*, *LOXC*, *LOXD*, and *LOXE*), *ADH2* and *HPL* are one of the key enzymes involving the synthesis fatty acid pathway-related volatiles in tomato fruits (26). As shown in Figure 3, there was no significant difference in the relative expression of *LOXA*, *LOXB* and *LOXE* genes among the fruits at different maturity stages stored at 20°C, while the relative expressions of *LOXC* and LOXD genes in tomatoes at P and R stages were remarkably higher compared with those at G and BR stages. When the fruits were at R stage, the relative expression of *LOXC* and *LOXD* genes was 882.4 or 241.4% higher than that of fruits at G stage. According to Chen et al. (27), *LOXC* was chloroplast-targeted and generated volatile C6 flavor compounds from both linoleic and linolenic acid, whilst *LOXD* could participate in the biosynthesis of jasmonic acid (27). Although no significant difference was observed in the *ADH2* gene expression of tomato fruits at four maturity stages without any treatments, the relative expression of *HPL* gene gradually reduced with the ripening of tomatoes (Figure 4). The expression level of *HPL* gene in fruits at R stage was on 41.2% that in those at G stage. Our data was similar to those obtained by Chen et al. (28) and Zhang et al. (29), they believed that the production of C6 aldehydes led to the changes in *HPL* gene expression (28, 29).

**FIGURE 5:**
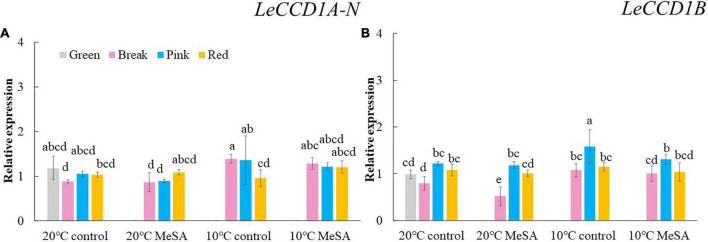
Effects of methyl salicylate (MeSA) pre-treatment on the relative expressions of *LeCCD1A-N* **(A)** and *LeCCD*1B **(B)** genes in tomato fruits stored at low temperature. Data are expressed as mean ± SD (*n* = 3), repeated measures one-way ANOVA followed by DMRT. Data marked with the same letter were no significant difference at *p* < 0.05.

**FIGURE 6:**
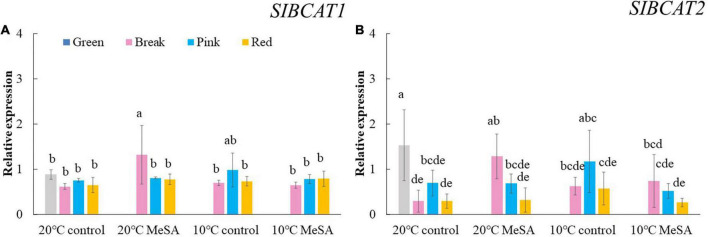
Effects of methyl salicylate (MeSA) pre-treatment on the relative expressions of *slBCAT1* **(A)** and *slBCAT2* **(B)** genes in tomato fruits stored at low temperature. Data are expressed as mean ± SD (*n* = 3), repeated measures one-way ANOVA followed by DMRT. Data marked with the same letter were no significant difference at *p* < 0.05.

Carotenoid cleavage can occur at any conjugated double bonds by *LeCCD*s to form an aldehyde or ketone (30). Two genes (*LeCCD1A-N* and *LeCCD1B*) were chosen in the present study, it was observed that the relative expression of *LeCCD* genes in tomato fruits decreased before the BK stage and then slowly increased (Figure 5). There was no significant difference in the *LeCCD* gene expression levels among the fruits at different maturity stages stored at 20°C, which was in accordance with the findings of Jing et al. (31). In terms of branched-chain amino acid pathway, *slBCAT*s are able to catalyze the initial step of degradation of leucine, isoleucine and valine to synthesize various flavor components (32). Figure 6 showed that the relative expressions of *slBCAT1* and *slBCAT2* genes in tomatoes of 20°C control group remarkably decreased with the ripening of fruits, which was probably brought about by the volatile production at different maturity stages (33). The *slBCAT1* or *slBCAT2* gene expression level in fruits at R stage stored at 20°C was 26.5 or 80.7% lower in comparison to that in those at G stage, respectively. In short, it seemed that the production of volatile compounds in tomato fruits was mainly influenced by their maturity through regulating fatty acid and branched-chain amino acid pathways.

### Effects of low temperature on the volatile profiles and key gene expressions of tomato fruits

It was shown in Table 2 that the total volatile contents in tomato fruits of 10°C control group were much lower compared to those of 20°C control group. When the fruits were at R stage, the total volatile content in fruits of 10°C control group was only 54.6% that a of 20°C control group. In line with previous studies (24), the formation of all types of aromatic compounds in tomatoes were considerably inhibited by low temperature treatment as well (Figure 1). The aldehyde, alcohol or ketone level in fruits at R stage stored at 20°C was 1.51-, 25.99-, or 1.47-fold that at 10°C, respectively.

Although there was no significant difference in the level of carotenoid pathway-related volatiles in fruits between 10°C control and 20°C control groups, the formation fatty acid pathway- and branched-chain amino acid pathway-related volatiles in tomatoes were suppressed by low temperature treatment (Figure 2). In fatty acid, carotenoid or branched-chain amino acid pathway, no significant difference was observed in the relative expressions of *LOXA*, *LOXE*, *ADH2*, *HPL, LeCCD1A-N*, *LeCCD1B, SlBCAT1*, and *SlBCAT2* genes in fruits at R stage between 20°C control group and 10°C control group (Figures 3–6). Whereas, low temperature treatment remarkably enhanced the expression levels of *LOXB* and *LOXC* genes as well as inhibited the *LOXD* gene expression level. At R stage, the relative expression of *LOXB*, *LOXC*, or *LOXD* gene in fruits of 10°C control group was 149.6, 209.5, or 6.2% that of 20°C control group, respectively. It seemed that low temperature treatment could greatly reduce the formation of fatty acid pathway-related volatiles through inhibiting the *LOXD* activity in tomatoes.

### Effects of methyl salicylate and methyl salicylate plus low temperature treatment on the volatile profiles and key gene expressions of tomato fruits

Methyl salicylate pre-treatment is a common postharvest technique to reduce the chilling injury of tomato fruits (34). Table 2 showed that the total volatile content of tomatoes in 20°C MeSA group was a little lower as compared to that in 20°C control group, which was consistent with the results of Wang et al. (6). At P stage, the aldehyde or alcohol level in fruits of 20°C MeSA group was 60.6 or 17.7% that of 20°C control group, respectively (Figure 1). In addition, the decrease of volatile compounds in low-temperature treated tomato fruits was effectively alleviated by MeSA pre-treatment. The total volatile content of fruits at R stage in 10°C MeSA group was 66.0% higher in comparison with that in 10°C control group, while the level of aldehydes, alcohols, or ketones in tomatoes at R stage in 10°C MeSA group was 1.51-, 16.69-, or 1.16-fold that in 10°C control group, respectively. Notably, MeSA pre-treatment significantly increased the *cis*-3-hexenal, hexanal and *trans*-2-hexenal levels in fruits subjected to low temperature. The enhancement of these aroma compounds was possibly associated with the direct action to *LOX* and β-oxidation pathways by regulating activities and expressions of *LOX*, *ADH*, *HPL*, and a series of other enzymes (35). Thereby, the effects of MeSA on the volatiles derived from different pathways and key gene expressions in low temperature-treated tomato fruits were further estimated in our research.

As shown in Figure 2, on the one hand, no significant difference was found in the levels of carotenoid pathway- and branched-chain amino acid pathway-related aroma compounds in fruits at R stage between 10°C MeSA group and 10°C control group. On the other hand, the biosynthesis of fatty acid pathway-related aroma compounds in tomatoes at P stage under 20°C was suppressed by MeSA pre-treatment. Whereas, the content of these components in tomatoes treated by MeSA greatly increased when the fruits turned red. The phenomenon was observed by Liu et al. (36) as well, they put forward that a high concentration of exogenous MeSA would increase endogenous salicylic acid concentration and induce cell damage in plants, which could subsequently result in the increased emission of fatty acid-derived compounds. Naturally, it was easily understood that the administration of MeSA in tomatoes stored at 10°C considerably inhibited the decrease of fatty acid pathway-related volatiles.

*LOX* pathway contributes to the formation of fruity note flavors in various fruits, including pears, kiwifruits and peaches (35, 37, 38). It was shown in Figure 3 that there was no significant difference in the expression levels of *LOXA*, *LOXB*, *LOXC*, and *LOXE* genes in fruits at R stage between 20°C MeSA group and 20°C control group. However, MeSA pre-treatment remarkably down-regulated the expression of *LOXD* gene in full red tomatoes stored under 20°C, which might be brought about by the protective effects of MeSA against the reduced membrane integrity in fruits (39). Regarding low temperature-treated tomato fruits at different maturity stages, MeSA pre-treatment had no significant effect on the expression of *LOXE* gene. In response to chilling injury, the relative expressions of *LOXA* and *LOXB* genes in low temperature-treated fruits at R stage were down-regulated by MeSA pre-treatment. Nevertheless, treating fruits with MeSA and low temperature greatly up-regulated the *LOXC* gene expression at BK stage as well as *LOXD* gene expression at R stage (Figures 3C,D). Specifically, the *LOX* gene expression level in fruits (BK stage) or *LOXD* gene expression level in fruits (R stage) of 10°C MeSA group was 160.9% or 80.8% higher compared to that of 10°C control group, respectively. A higher expression of *LOX* genes could promote the formation of straight-chain alcohols, esters and lactones (34). Thus, the results obtained in volatile analysis were confirmed by those of qRT-PCR experiment.

*ADH2* plays crucial roles in the reaction of aldehydes into alcohols (40), while *HPL* is essential for the green note flavors on account of its effects on the biosynthesis of aldehydes and oxoacids (41). As shown in Figure 4, MeSA pre-treatment had no remarkable impact on the *ADH2* and *HPL* expression levels in low temperature-treated tomatoes at P and R stages. We hypothesized that the increase of alcohols and esters in volatile compounds of tomatoes at R stage in 10°C MeSA group was probably caused by the up-regulation effects of MeSA on the relative expression of alcohol o-acyltransferase rather than *ADH2* (42). *LeCCDs* and *slBCAT*s are involved in the metabolism carotenoid pathway- and branched-chain amino acid pathway-related volatiles. Figures 5, 6 showed that there was no significant difference in the relative expressions of LeCCD1A-N, LeCCD1B, slBCAT1 and slBCAT2 genes between 20°C MeSA group and 20°C control group, which was matched with the data of volatile analysis. Overall, MeSA pre-treatment might avoid the loss of volatiles compounds in tomato fruits stored at low temperature through activating the fatty acid pathway.

## Conclusion

In the present investigation, the effects of MeSA pre-treatment on the volatile profile and volatile biosynthesis pathways of low temperature-treated tomatoes were studied. Our results indicated that low temperature treatment could inhibit the biosynthesis of aromatic components in tomato fruits at P and R stages. Whereas, MeSA pre-treatment was able to effectively alleviate the loss of volatile compounds in tomato fruits stored at 10°C, especially aldehydes, alcohols and ketones. Notably, the level of fatty acid pathway-related volatiles (including *cis*-3-hexenal, hexanal and *trans*-2-hexenal) in full red fruits of 10°C MeSA group was significantly higher as compared to that of 10°C control group, although MeSA pre-treatment had no remarkable effect on the formation of carotenoid pathway- and branched-chain amino acid pathway-related flavor components in tomatoes treated by low temperature. Based on the data of qRT-PCR analysis, MeSA plus low temperature treatment remarkably up-regulated the *LOXC* or *LOXD* gene expression in fruits at BK or R stage compared to low temperature treatment, while there was no significant difference in the relative expressions of *ADH2*, *HPL*, *LeCCD*, and *slBCAT* genes between 20°C MeSA group and 20°C control group. In a nutshell, the loss of aromatic compounds in low temperature-treated tomato fruits could be suppressed by MeSA pre-treatment through activating the fatty acid pathway.

## Data availability statement

The original contributions presented in this study are included in the article/supplementary material, further inquiries can be directed to the corresponding author.

## Author contributions

XZ: resources (lead), writing—original draft (lead), and funding acquisition (lead). LW and JHZ: supervision (supporting). YF, YL, JLZ, and RC: writing—review and editing (supporting). JL: conceptualization (lead), funding acquisition (lead), supervision (lead), and writing—review and editing (lead). All authors contributed to the article and approved the submitted version.

## Footnotes

1
https://mafft.cbrc.jp/alignment/server/
